# Supplementary material for: Predicting visual function by interpreting a neuronal wiring diagram
Source: Nature. 2024 Oct 2;634(8032):113–23. doi: 10.1038/s41586-024-07953-5 (PMC11446822; doi:10.1038/s41586-024-07953-5)
Supplement: Supplementary file 6 — CRF and ERF predictions for individual TmY4 and TmY9 cells. Analogous to Supplementary Data 3, but for TmY target types. Shown are the top four monosynaptic pathways, the strongest pathway passing through each of the top ten intermediary types (ranking from Extended Data Fig. 7), and the trisynaptic pathway Tm1–TmY–Dm3–TmY (see the section entitled Prediction of spatial normalization). [file 41586_2024_7953_MOESM6_ESM.zip › DataS4/TmY9q/720575940627286044.pdf]

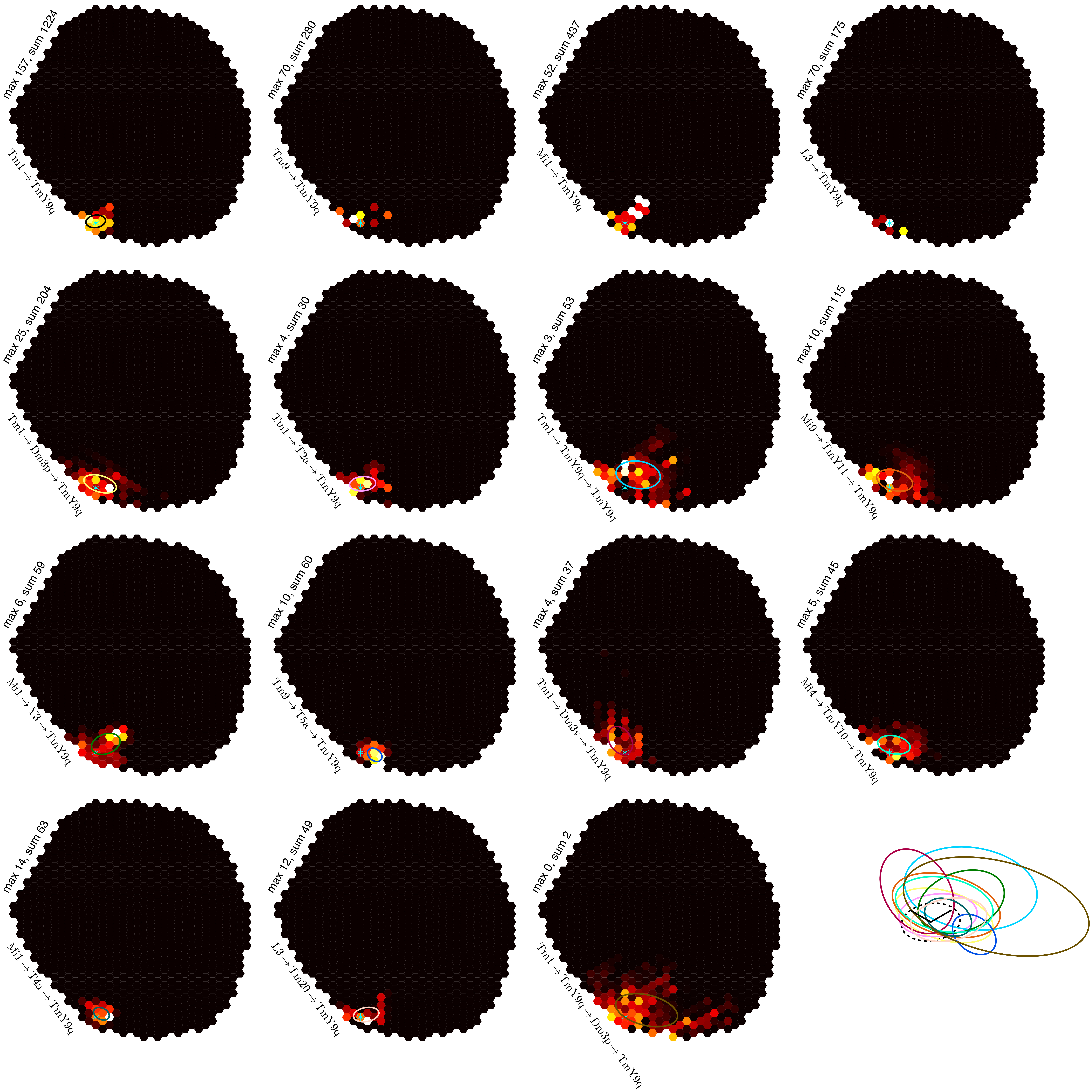

max 157, sum 1224
Tm1 → TmY9q
max 70, sum 280
Tm9 → TmY9q
max 52, sum 437
Mi1 → TmY9q
max 70, sum 175
L3 → TmY9q
max 25, sum 204
Tm1 → Dm3p → TmY9q
max 4, sum 30
Tm1 → T2a → TmY9q
max 3, sum 53
Tm1 → TmY9q → TmY9q
max 10, sum 115
Mi9 → TmY11 → TmY9q
max 6, sum 59
Mi1 → Y3 → TmY9q
max 10, sum 60
Tm9 → T5a → TmY9q
max 4, sum 37
Tm1 → Dm3v → TmY9q
max 5, sum 45
Mi4 → TmY10 → TmY9q
max 14, sum 63
Mi1 → T4a → TmY9q
max 12, sum 49
L3 → Tm20 → TmY9q
max 0, sum 2
Tm1 → TmY9q → Dm3p → TmY9q
